# Interim 2018/19 influenza vaccine effectiveness: six European studies, October 2018 to January 2019

**DOI:** 10.2807/1560-7917.ES.2019.24.1900121

**Published:** 2019-02-21

**Authors:** Esther Kissling, Angela Rose, Hanne-Dorthe Emborg, Alin Gherasim, Richard Pebody, Francisco Pozo, Ramona Trebbien, Clara Mazagatos, Heather Whitaker, Marta Valenciano, European IVE group

**Affiliations:** 1 EpiConcept, Paris, France; 2 These authors contributed equally to the study and manuscript writing; 3 Department of Infectious Disease Epidemiology and Prevention, Statens Serum Institut, Copenhagen, Denmark; 4 National Epidemiology Centre, Institute of Health Carlos III, Madrid, Spain; CIBER de Epidemiología y Salud Pública (CIBERESP), Institute of Health Carlos III, Madrid, Spain; 5 Public Health England, London, United Kingdom; 6 National Centre for Microbiology, National Influenza Reference Laboratory, WHO-National Influenza Centre, Institute of Health Carlos III, Madrid, Spain; 7 Department of Virus and Microbiological Special diagnostics, National Influenza Center, Statens Serum Institut, Copenhagen, Denmark; 8 European Influenza Vaccine Effectiveness (IVE) group members are listed at the end of the article

**Keywords:** influenza, vaccine effectiveness, multicentre study, test-negative design, Europe, vaccination, vaccines and immunisation

## Abstract

Influenza A(H1N1)pdm09 and A(H3N2) viruses both circulated in Europe in October 2018–January 2019. Interim results from six studies indicate that 2018/19 influenza vaccine effectiveness (VE) estimates among all ages in primary care was 32–43% against influenza A; higher against A(H1N1)pdm09 and lower against A(H3N2). Among hospitalised older adults, VE estimates were 34–38% against influenza A and slightly lower against A(H1N1)pdm09. Influenza vaccination is of continued benefit during the ongoing 2018/19 influenza season.

Seasonal influenza vaccine is recommended in all European Union (EU) countries for older people and others at increased risk of severe influenza and its complications, including those with chronic diseases [1]. In the United Kingdom (UK), incremental introduction of a universal childhood influenza vaccination programme began in 2013/14 [2].

The World Health Organization (WHO) recommendations for trivalent influenza vaccine strains for the 2018/19 northern hemisphere influenza season included an A/Michigan/45/2015 (H1N1)pdm09-like virus, an A/Singapore/INFIMH-16–0019/2016 (H3N2)-like virus and a B/Colorado/06/2017-like virus from the B/Victoria lineage [3].

The early 2018/19 influenza season in Europe was characterised by both influenza A virus subtypes circulating widely. There was co-circulation in some countries, with others reporting dominance of either A(H1N1)pdm09 or A(H3N2) viruses. The season started late in most countries compared with previous seasons, with few influenza B viruses detected in the WHO European Region [4]. Since the 2008/09 season, the UK, Denmark, Spain, and several other EU countries conducting multicentre studies, have participated in I-MOVE (Influenza – Monitoring Vaccine Effectiveness in Europe), a network measuring influenza vaccine effectiveness each season.

We summarise interim 2018/19 season influenza vaccine effectiveness (VE) estimates from four single-country and two multi-country studies, including both outpatient and hospital settings, in order to help guide influenza prevention and control measures for the rest of the 2018/19 season.

## Study setting

**Figure 1:**
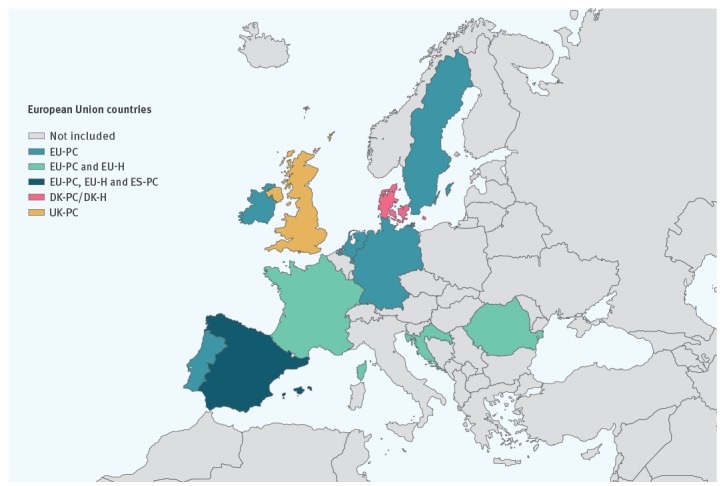
European Union countries contributing to the interim influenza vaccine effectiveness results, influenza season 2018/19 (n  = 11)

The primary care (PC) setting studies were conducted in Denmark (DK-PC), Spain (ES-PC), the UK (UK-PC) and via the European Union (EU) I-MOVE multi-country network (EU-PC). The hospital setting (H) studies were undertaken in Denmark (DK-H) and via the EU I-MOVE multi-country network (EU-H) (Figure 1).

DK-PC/DK-H: Denmark primary care and hospital studies; ES-PC: Spain primary care study; EU-H: European hospital-based multi-country I-MOVE study; EU-PC: European primary care-based multi-country I-MOVE study; I-MOVE: Influenza - monitoring of vaccine effectiveness in Europe; UK: United Kingdom; UK-PC: UK primary care study.

## Study design and estimation of vaccine effectiveness

The methods of these six studies are described in detail elsewhere [5-9]. All six studies used a test-negative case control design, with differences between studies in how data were collected and how patients were selected (Table 1) [10]. Briefly, individuals presenting to participating healthcare settings with symptoms of influenza-like illness (ILI) (primary care settings) or severe acute respiratory infection (hospital settings) were swabbed. These samples were then tested by reverse transcription (RT)-PCR for influenza virus. Patients with positive results were classified as cases (by influenza virus (sub)type), and those with negative results as controls.

**Table 1:** Summary characteristics of the included influenza vaccine effectiveness studies, Europe, interim influenza season 2018/19 (n  = 23,007)

	DK-PC	ES-PC	EU-PC	UK-PC	DK-H	EU-H
Study period	1 November 2018– 31 January 2019	5 November 2018–18 January 2019	21 October 2018–23 January 2019	1 October 2018–18 January 2019	1 November 2018–31 January 2019	5 December 2018–18 January 2019
Setting	Primary care	Primary care	Primary care	Primary care	Hospital	Hospital
Location	Denmark	Spain: Sentinel networks in 16 of 19 regions	Croatia, France, Germany, Ireland, the Netherlands, Portugal, Romania, Spain (five regions) and Sweden	England, Scotland, Northern Ireland and Wales	Denmark	11 hospitals in: Croatia, France, Spain and Romania
Study design	TND	TND	TND	TND	TND	TND
Data source	Data linkage of Danish Microbiology Database, the Danish Vaccination Register and the Danish National Discharge Register	Sentinel physicians and laboratory^a^	Sentinel physicians and laboratory^a^	Sentinel physicians and laboratory	Data linkage of Danish Microbiology Database, the Danish Vaccination Register and the Danish National Discharge Register	Hospital charts, vaccine registers, interviews with GPs, laboratory
Age groups of study population	All ages	≥ 6 months	≥ 6 months	All ages	All ages	≥ 65 years
Case definition	Sudden onset of symptoms with fever, myalgia and respiratory symptoms	EU ILI	EU ILI	ILI: Patient presenting in primary care with an acute respiratory illness with physician diagnosed fever with onset in previous 7 days	SARI: Sudden onset of symptoms with fever, myalgia and respiratory symptoms among hospitalised patients	EU SARI
Selection of patients	At practitioner's judgement	Systematic	Systematic	At practitioner's judgement	At practitioner's judgement	Exhaustive
Vaccine types used nationally or in the study^b^	In the study among controls: 21% QIV, 79% TIV	The following vaccine types are available in Spain: TIV, adjuvanted TIV, QIV	In the study among controls: 44% QIV, 29% TIV, 23% unavailable, 3% adjuvanted TIV, 1% LAIV4	Healthy children 2–11 years of age: LAIV4; At risk children < 18 years of age: QIV; Adults 18–64 years of age: QIV; Adults ≥ 65 years: mainly adjuvanted TIV with some differences across UK countries.	In the study among controls: 18% QIV, 82% TIV	In the study among controls: 53% TIV, 35% adjuvanted TIV, 6% QIV and 6% unknown
Variables of adjustment	Age group, sex, presence of chronic conditions, number of hospitalisations in previous year, calendar time as month (Nov-Jan)	For all ages: Age (RCS), onset date (RCS), sex, chronic conditions, region; For target groups: Age (RCS), onset date (RCS), sex, region	Age (modelled as RCS or age group depending on analysis), sex, presence of any chronic condition associated with influenza vaccination recommendation, onset date (RCS) and study site	Age group, sex, onset month, pilot area for child vaccination programme, surveillance scheme, risk group	Age group, sex, presence of chronic conditions, number of hospitalisations in previous year, calendar time as month (November–January)	Age, sex, presence/number of chronic conditions, onset date (modelled as RCS or categorical depending on analysis) and study site

DK-H: Denmark hospital study; DK-PC: Denmark primary care study; ES-PC: Spain primary care study; EU: European Union; EU-H: European hospital multicentre I-MOVE study; EU-PC: European primary care multicentre I-MOVE study; GP: general practitioner; ILI: influenza-like illness; I-MOVE: Influenza - monitoring of vaccine effectiveness in Europe; LAIV4: quadrivalent live attenuated influenza vaccine; LRI: lower respiratory infection; QIV: quadrivalent inactivated influenza vaccines; RCS: restricted cubic spline; SARI: severe acute respiratory infection; TND: test-negative design; UK: United Kingdom; UK-PC: UK primary care study.

^a^122 of 805 physicians included in ES-PC were also included in EU-PC.

^b^Vaccines were egg-propagated, non-adjuvanted and administered intramuscularly unless otherwise specified.

Patients were defined as vaccinated with the 2018/19 influenza vaccine if they were vaccinated at least 14 or 15 days (depending on the study) before symptom onset. Patients were excluded if they were vaccinated fewer than 14 or 15 days before symptom onset, or if the date of vaccination was unknown.

In eight EU-PC countries, DK-PC and DK-H, all or a random sample of influenza virus-positive specimens were selected for sequencing (haemagglutinin genome segment and/or whole genome). In ES-PC, in regions not included in EU-PC, an ad hoc sample of influenza viruses was sequenced. In UK-PC, all influenza viruses with sufficient genetic material (Ct value < 31) were sequenced, as well as all viruses derived from vaccinated cases. Sequencing results in Denmark were combined for both studies (DK-PC and DK-H).

We computed VE by comparing the odds of vaccination between cases and controls (VE = (1 – odds ratio (OR)) x 100%). All studies used logistic regression to adjust their VE for measured confounding variables (Table 1). Study-specific VE was estimated overall and where possible, by age group and target population (as defined locally in the various studies and study sites) against influenza A overall, A(H1N1)pdm09 and A(H3N2). If the number of cases (or controls if lower) per parameter was less than 10, a sensitivity analysis was performed using Firth’s method of penalised logistic regression to assess small sample bias [11,12]. Where exposed case numbers were zero, exact logistic regression was used.

## Results

From 1 October 2018 to 31 January 2019, the total number of patients included in each study for the influenza A analysis in primary care settings was: DK-PC (11,910; 2,807 cases), ES-PC (1,204; 476 cases), UK-PC (936; 177 cases), EU-PC (2,079; 478 cases). In the hospital settings numbers were: DK-H (6,520; 653 cases), EU-H (298; 67 cases).

**Figure 2:**
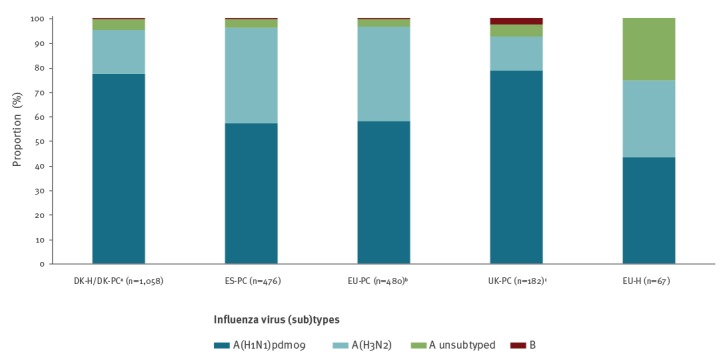
Proportion of influenza virus (sub)types by study, 11 European countries, interim influenza season 2018/19 (n = 2,263)

DK-H: Denmark hospital study; DK-PC: Denmark primary care study; ES-PC: Spanish primary care study; UK: United Kingdom; UK-PC: UK primary care study; EU-H: European hospital multicentre I-MOVE study; EU-PC: European primary care multicentre I-MOVE study; I-MOVE: Influenza - monitoring of vaccine effectiveness in Europe.

^a^Based on data obtained at the National Influenza Centre, Denmark (cannot be divided into hospital and primary care separately). A further 2,404 influenza A cases from other laboratories were included in the vaccine effectiveness analysis, representing all clinical microbiological laboratories in Denmark.

^b^62 of influenza A(H1N1)pdm09 and 46 of A(H3N2) cases were also in ES-PC.

^c^Includes one A(H1N1)pdm09 and B co-infection.

In all studies combined, 99.5% (2,252/2,263) of cases were influenza A virus-positive. The proportion of influenza A viruses subtyped in the DK-H/DK-PC, ES-PC, EU-PC and UK-PC was ≥ 95% and in the EU-H it was 75%. Of influenza viruses subtyped, 58–60% were influenza A(H1N1)pdm09 viruses in ES-PC, EU-PC and EU-H; while this proportion was > 80% in DK-PC/DK-H and UK-PC (Figure 2).

### Influenza A overall

#### Primary care settings

In primary care settings among all ages, VE against laboratory-confirmed influenza A ranged between 32% (95% confidence interval (CI): -25 to 63) in ES-PC and 43% in UK-PC and in EU-PC (95% CI: 3 to 67 and 6 to 65, respectively). The VE against influenza A among patients aged 18–64 years ranged from 32% (95% CI: -31 to 65) in the EU-PC to 55% (95% CI: 44 to 64) in the DK-PC study. In children aged 2–17 years in UK-PC, the VE of quadrivalent live attenuated influenza vaccines (LAIV4) was 80% (95% CI: -54 to 97) (Table 2). Among target groups for influenza vaccination, VE was 59% in both ES-PC and EU-PC (95% CI: - 1 to 83 and 32 to 78, respectively).

**Table 2:** Adjusted seasonal vaccine effectiveness against laboratory-confirmed influenza A, A(H1N1)pdm09 and A(H3N2), by age group, target group for vaccination and study, 11 European countries, interim influenza season 2018/19

Influenza type/subtype and study site	Setting	Study population	Cases			Controls			Adjusted VE	95% CI
			All	Vacc	%	All	Vacc	%		
**Influenza A**										
DK-PC	PC	All ages	2,807	342	12	9,103	1,925	21	38	29 to 46
		18–64 years	1,509	112	7	4,298	633	15	55	44 to 64
		≥ 65 years	398	218	55	2,115	1,183	56	4	-19 to 23
ES-PC	PC	All ages	476	32	7	728	57	8	32	-25 to 63
		Target group	85	19	22	145	42	29	59	-1 to 83
EU-PC	PC	All ages	478	35	7	1,601	160	10	43	6 to 65
		0–17 years	142	4	3	570	17	3	58	-111 to 92
		18–64 years	296	19	6	846	62	7	32	-31 to 65
		Target group^a^	123	25	20	412	124	30	59	32 to 78
UK-PC	PC	All ages	177	31	18	819	224	27	43	3 to 67
		2–17 years (LAIV4)	27	2	NC	119	25	21	80	-54 to 97
		2–17 years (LAIV4 or TIV)	28	3	NC	123	29	24	67	-80 to 94
		18–64 years	135	20	15	440	90	20	37	-20 to 67
DK-H	Hospital	All ages	653	187	29	5,867	2,321	40	38	24 to 49
		18–64 years	272	46	17	1,894	455	24	39	14 to 57
		≥ 65 years	297	138	46	3,174	1,827	58	34	16 to 48
EU-H	Hospital	≥ 65 years	67	30	45	231	144	62	38	-12 to 65
**Influenza A(H1N1)pdm09**										
DK-PC	PC	All ages	980	72	7	9,103	1,925	21	55	41 to 65
		18–64 years	573	32	6	4,298	633	15	66	51 to 77
		≥ 65 years	72	38	53	2,115	1,183	56	0	-61 to 38
ES-PC	PC	All ages	272	14	5	728	57	8	45	-20 to 75
		Target group^a^	49	8	NC	145	42	29	61	-22 to 88
EU-PC	PC	All ages	272	10	4	1,381	153	11	71	38 to 86
		18–64 years	178	5	3	736	59	8	75	27 to 91
UK-PC	PC	All ages	143	20	14	819	224	27	57	20 to 77
		2–17 years (LAIV)^b^	23	0	NC	123	29	24	87	4 to 100
		2–17 years (LAIV4 or TIV) ^b^	23	0	NC	123	29	24	89	19 to 100
		18–64 years	111	16	14	440	90	20	39	-23 to 69
DK-H	Hospital	All ages	228	57	25	5,867	2,321	40	40	17 to 57
		18–64 years	110	17	15	1,894	455	24	49	13 to 70
		≥ 65 years	85	38	45	3,174	1,827	58	37	3 to 60
EU-H	Hospital	≥ 65 years	28	13	NC	177	112	63	29	-75 to 71
**Influenza A(H3N2)**										
DK-PC	PC	All ages	136	24	18	9103	1,925	21	24	-22 to 55
		18–64 years	78	6	8	4,298	633	15	48	-23 to 78
ES-PC	PC	All ages	186	17	9	728	57	8	-9	-147 to 52
EU-PC	PC	All ages	179	21	12	1,437	134	9	-3	-100 to 47
UK-PC	PC	All ages	25	9	NC	819	224	27	-39	-305 to 52
EU-H	Hospital	≥ 65 years	20	9	NC	198	127	64	47	-48 to 81

CI: confidence interval; DK-PC: Denmark primary care study; DK-H: Denmark hospital study; ES-PC: Spain primary care study; EU-H: European hospital multicentre I-MOVE study; EU-PC: European primary care multicentre I-MOVE study; I-MOVE: Influenza - monitoring of vaccine effectiveness in Europe; LAIV4: quadrivalent live attenuated influenza vaccine; NC: Not calculated (percentages not shown where denominators < 60); TIV: trivalent live attenuated vaccines; UK: United Kingdom; UK-PC: UK primary care study; Vacc: vaccinated; VE: vaccine effectiveness.

^a^Groups targeted by seasonal influenza vaccination as defined locally in the studies and study sites.

^b^While the modal estimate of VE is 100% due to no exposed cases, the point estimates given are from exact logistic regression in Stata with adjustment for month and age where the median estimate is used from the conditional likelihood distribution.

Study sites included in EU-H analysis for influenza A: Croatia, France, Romania and Spain. For analysis against influenza A(H1N1)pdm09: Romania and Spain only. For analysis against influenza A(H3N2): Romania and Spain only.

Study sites included in EU-PC analysis for influenza A: Croatia, France, Germany, Ireland, the Netherlands, Portugal, Romania, Spain and Sweden. For analysis against influenza A(H1N1)pdm09: France, Germany, Ireland, the Netherlands, Portugal, Romania, Spain and Sweden are included. For analysis against influenza A(H3N2): France, Germany, the Netherlands, Portugal, Romania, Spain and Sweden are included.

#### Hospital settings

VE against laboratory-confirmed hospitalised influenza A among all ages in DK-H was 38% (95% CI: 24 to 49) and in patients aged 65 years and older, VE was 34% (95% CI: 16 to 48) in DK-H and 38% (95% CI: - 12 to 66) in EU-H.

### Influenza A(H1N1)pdm09

#### Primary care settings

In the primary care studies, VE against laboratory-confirmed influenza A(H1N1)pdm09 among all ages ranged from 45% (95% CI: -20 to 75) in ES-PC to 71% (95% CI: 38 to 86) in EU-PC.

In UK-PC, the VE of LAIV4 among children aged 2–17 years was 87% (95% CI: 4 to 100). Among patients aged 18–64 years, VE was between 39% (95% CI: -23 to 69) and 75% (95% CI: 27 to 91) in UK-PC and EU-PC, respectively. VE among those aged 65 years and older was 0% (95% CI: - 61 to 38) in the DK-PC study.

#### Hospital settings

In hospital-based studies among patients aged 65 years and older, VE was 29% (95% CI: - 75 to 71) in EU-H and 37% (95% CI: 3 to 60) in the DK-H study (Table 2). VE among those aged 18–64 years was 49% (95% CI: 13 to 70; DK-H).

#### Virological results

All 265 A(H1N1)pdm09 viruses sequenced belonged to clade 6B.1 (A/Michigan/45/2015) (Table 3). Among 240 viruses (91%) with information on substitutions in the haemagglutinin gene, all harboured additional substitutions of S74R (except one of the 83 sequenced in DK-H/DK-PC), S164T and I295V, and most of them also included the substitution S183P. The proportion of other substitutions identified (T120A, N129D, E235D and K302T) differed by study (Table 3). None of these substitutions involve a change in potential glycosylation sites.

**Table 3:** Influenza viruses characterised by clade, amino acid substitutions and study site, 11 European countries, interim influenza season 2018/19 (n = 428)

	Clade	DK-H/DK-PC^a^		ES-PC^b^		EU-PC^c,d^		UK-PC^d^	
		n	%	n	%	n	%	n	%
**Total influenza A(H1N1)**		**n = 820**		**n = 272**		**n = 272**		**n = 152**	
Sequenced		83	100	78	100	79	100	25	NC
A/Michigan/45/2015	6B.1 / Substitutions not available	0	0	0	0	0	0	25	NC
A/Michigan/45/2015 ^e^	6B.1 / None of the below	2	2	3	4	4	5	NA	NA
A/Michigan/45/2015 ^e^	6B.1 / T120A	29	35	8	10	2	3	NA	NA
A/Michigan/45/2015 ^e^	6B.1 / N129D	25	30	31	40	50	63	NA	NA
A/Michigan/45/2015 ^e^	6B.1 / E235D	0	0	19	24	3	4	NA	NA
A/Michigan/45/2015 ^e^	6B.1 / K302T	27	33	17	22	15	19	NA	NA
A/Michigan/45/2015 ^e^	6B.1 / T120A + K302T	0	0	0	0	1	1	NA	NA
**Total influenza A(H3N2)**		**n = 187**		**n = 186**		**n = 179**		**n = 34**	
Sequenced		30	NC	79	100	52	NC	2	NC
A/Alsace/1746/2018 ^f^	3C.2a1b	29	NC	31	39	34	NC	2	NC
A/Switzerland/8060/2017 ^f^	3C.2a2	1	NC	0	0	1	NC	0	NC
A/Cote d’Ivoire/544/2016 ^f^	3C.2a3	0	NC	4	5	7	NC	0	NC
A/England/538/2018 ^f^	3C.3a	0	NC	44	56	10	NC	0	NC

DK-PC: Denmark primary care study; DK-H: Denmark hospital study; ES-PC: Spain primary care study; EU-PC: European primary care multicentre I-MOVE study; I-MOVE: Influenza - monitoring of vaccine effectiveness in Europe; NA: not available; NC: not calculated (percentages not shown where denominators < 60); UK: United Kingdom; UK-PC: UK primary care study.

^a^DK-H and DK-PC are combined; sequence information is based on influenza-positive samples received for surveillance at the National Influenza Center Denmark from week 41/2018 and 03/2019.

^b^Specimens sequenced from Spain originate from the entire National Influenza Surveillance System in weeks 45/2018–03/2019.

^c^18 specimens from ES were also included in EU-PC data (12 A/Alsace/1746/2018, 4 A/Cote d’Ivoire/544/2016, two A/Michigan/45/2015).

^d^At time of publishing, not all specimens from the study period were processed.

^e^All include additional substitutions S74R, S164T and I295V, and most also include S183P substitutions.

^f^Representative strains for the clades.

### Influenza A(H3N2)

#### Primary care and hospital settings

In primary care studies, among all ages, VE against influenza A(H3N2) ranged from - 39% (95% CI: - 305 to 52) in UK-PC to 24% (95% CI: - 22 to 55) in DK-PC. VE among patients aged 65 years and older hospitalised for influenza A(H3N2) was 47% (95% CI: - 48 to 81) in EU-H (Table 2).

#### Virological results

Of 163 influenza A(H3N2) viruses sequenced, 59% (n = 96) belonged to genetic clade 3C.2a1b, 33% (n = 54) to 3C.3a, 7% (n = 11) to 3C.2a3 and 1% (n = 2) to 3C.2a2 (Table 3). Both A(H3N2) viruses sequenced in UK-PC, 29/30 A(H3N2) viruses sequenced in DK-H/DK-PC, 34/52 in EU-PC and 31/79 in ES-PC belonged to clade 3C.2a1b. Of 79 A(H3N2) viruses sequenced in ES-PC, 44 (56%) belonged to clade 3C.3a.

#### Sensitivity analyses

Sensitivity analyses for small sample size gave similar results (absolute difference range 1–9%).

## Discussion

Interim results from six established influenza VE studies across Europe for the 2018/19 season indicate that VE against laboratory-confirmed influenza A ranged between 32% and 43% among all ages in primary care and hospital settings and was 59% in the target groups for vaccination.

Against influenza A(H1N1)pdm09, VE point estimates among all ages ranged from 40% to 71%, and were lower among older adults in DK-PC, DK-H and EU-H, ranging from 0% to 37%. Against influenza A(H3N2), the results of three of four primary care studies suggest that the vaccine was not effective among all ages combined. The VE point estimate against A(H3N2) was higher among older adults in EU-H and among 18–64-year-olds in DK-PC (47% and 48%, respectively). The low number of A(H3N2) cases in all studies resulted in less precise VE estimates against A(H3N2) than against A(H1N1)pdm09.

The influenza A(H1N1)pdm09 VE point estimates among all ages in EU-PC, among adults in DK-PC and EU-PC and among children in the UK-PC were similar to 2018/19 interim VE estimates in Canada [13]. For all ages combined, point estimates for this subtype for ES-PC and DK-H were similar to those recently reported from the United States (US) [14]. In UK-PC, the LAIV4 VE point estimate was high against influenza A(H1N1)pdm09, although sample size was very small. This suggests that the A(H1N1)pdm09 LAIV4 vaccine virus strain change from A/Bolivia/559/2013 to A/Slovenia/2903/2015 that took place after the 2016/17 season may have improved vaccine performance against circulating strains in 2018/19. Compared with 2017/18 interim season estimates in studies where influenza A(H1N1)pdm09 VE results were available, the 2018/19 adjusted VE against influenza A(H1N1)pdm09 was similar in the 18–64 years age group in DK-PC (66% vs 60%, respectively, noting that in 2017/18 the setting in Denmark was primary care and hospital combined) and among all ages in EU-PC (71% vs 68%, respectively). VE was lower among those aged  65 years and older in DK-PC, but similar in the DK-H study.

The genetic diversity observed in the ongoing 2018/19 season did not seem to affect the VE against influenza A(H1N1)pdm09 in most groups and studies. To date, all A(H1N1)pdm09 viruses characterised in Europe were antigenically similar to the vaccine virus [15]. The lower VE among those aged 65 and older in DK-PC may be explained by small sample size, but needs further investigation.

As observed in the 2017/18 season, the 2018/19 interim primary care results suggest that VE against medically attended laboratory-confirmed influenza A(H3N2) was low or non-existent although, due to small sample size, these interim 2018/19 results need to be confirmed by the end-of-season results. End-of-season clade-specific VE results may help us understand whether regional differences in circulating clades of A(H3N2) viruses explain the difference in VE in DK-PC compared with all other primary care studies. Adaptation/alteration of the vaccine seed virus during propagation in eggs, impacting antigenicity, may have been an important explanation for low VE against influenza A(H3N2) in recent and current seasons [16].

The late start of the season resulted in small sample sizes and low precision of many VE estimates, which presents a limitation in this interim analysis. We thus conducted a sensitivity analysis to address potential small sample bias arising from this. Further limitations potentially present in all observational studies include residual confounding and bias.

Vaccination continues to be the most effective preventive measure against influenza and uptake of the 2018/19 influenza vaccines should still be promoted in countries with ongoing influenza virus circulation in line with national guidelines and recommendations. Our results further support the need for effective interventions against influenza A(H3N2) across all age groups. In the UK, the Joint Committee on Vaccination and Immunisation has recently advised the use of cell-grown influenza vaccine that will be licensed for the 2019/20 season for older children and adults in the UK [17]. In addition, given the observed non-effectiveness of the A(H3N2) component of the current vaccine in previous seasons, in settings with influenza A(H3N2) virus circulation, prophylactic and prompt therapeutic use of neuraminidase inhibitors is important to help prevent severe outcomes, irrespective of vaccination status [18].

The Global Influenza VE (GIVE) Collaboration reports on the effectiveness of influenza vaccine in previous and current influenza seasons. Interim VE results presented here were included in the February 2019 GIVE report to help inform the WHO vaccine strain selection committee meeting on 18–21 February 2019 in Bejing. For the 2019/20 northern hemisphere trivalent vaccine, this selection committee recommended to include an A/Brisbane/02/2018 (H1N1)pdm09-like virus and a B/Colorado/06/2017-like virus (B/Victoria/2/87 lineage) [19]. For the quadrivalent vaccine WHO recommended an additional B/Phuket/3073/2013-like virus (B/Yamagata/16/88 lineage). The recommendation for the A(H3N2) component will be postponed until 21 March 2019, due to changes in the proportions of genetically and antigenically diverse A(H3N2), notably an increase in clade 3C.3a in several geographic regions.

End-of-season VE and antigenic studies will provide insight into age- and study-specific variation in VE estimates. In addition, monitoring effectiveness of the 2019 southern hemisphere influenza vaccine against influenza viruses and their genetic diversity will be important to prepare for the next influenza season in the northern hemisphere.

## European IVE group

Croatia

EU-PC + EU-H studies:

Sanja Kurecic Filipovic, Croatian Institute of Public Health, Zagreb

Vesna Visekruna-Vucina, Croatian Institute of Public Health, Zagreb

Iva Pem Novosel, Croatian Institute of Public Health, Zagreb

Zvjezdana Lovric, Croatian Institute of Public Health, Zagreb

Goranka Petrović, Croatian Institute of Public Health, Zagreb

Vladimir Draženović, Croatian Institute of Public Health, Virology Department, Zagreb

EU-H study:

Adriana Vince, University Hospital for Infectious Diseases, Zagreb

Antea Topić, University Hospital for Infectious Diseases, Zagreb

Jelena Budimir Mihalić, University Hospital for Infectious Diseases, Zagreb

Eva Huljev, University Hospital for Infectious Diseases, Zagreb

Boris Lukšić, Clinical Hospital Centre Split

Svjetlana Karabuva, Clinical Hospital Centre Split

Mihaela Čikeš, Clinical Hospital Centre Split

Denmark

DK study:

Hanne-Dorthe Emborg, Department of Infectious Disease Epidemiology and Prevention, Statens Serum Institut, Copenhagen

Ramona Trebbien, Department of Virus and Microbiological Special diagnostics, National Influenza Center, Statens Serum Institut, Copenhagen

Ida Glode Helmuth, Department of Infectious Disease Epidemiology and Prevention, Statens Serum Institut, Copenhagen

Tyra Grove Krause, Department of Infectious Disease Epidemiology and Prevention, Statens Serum Institut, Copenhagen

France

EU-PC and EU-H studies:

Bruno Lina, Laboratoire de Virologie, CNR des virus des infections respiratoires, Institut des Agents Infectieux, Groupement Hospitalier Nord des HCL, Lyon, France; Laboratoire Virpath, CIRI Inserm U1111, CNRS 5308, ENS, UCBL, Faculté de Médecine LYON Est, Université de Lyon, Lyon.

EU-PC study:

Alessandra Falchi, Laboratoire de Virologie, Université de Corse-Inserm, Corte.

Ana-Maria Vilcu, Sorbonne Université, INSERM, Institut Pierre Louis d’Epidémiologie et de Santé Publique (IPLESP UMRS 1136), F75012 Paris

Cécile Souty, Sorbonne Université, INSERM, Institut Pierre Louis d’Epidémiologie et de Santé Publique (IPLESP UMRS 1136), Paris

Thierry Blanchon, Sorbonne Université, INSERM, Institut Pierre Louis d’Epidémiologie et de Santé Publique (IPLESP UMRS 1136), Paris

Titouan Launay, Sorbonne Université, INSERM, Institut Pierre Louis d’Epidémiologie et de Santé Publique (IPLESP UMRS 1136), Paris

Sylvie van der Werf, Unité de Génétique Moléculaire des Virus à ARN, UMR 3569 CNRS, Université Paris Diderot SPC, Institut Pasteur, Paris; CNR des virus des infections respiratoires, Institut Pasteur, Paris

Vincent Enouf, Unité de Génétique Moléculaire des Virus à ARN, UMR 3569 CNRS, Université Paris Diderot SPC, Institut Pasteur, Paris; CNR des virus des infections respiratoires, Institut Pasteur, Paris.

Sylvie Behillil, Unité de Génétique Moléculaire des Virus à ARN, UMR 3569 CNRS, Université Paris Diderot SPC, Institut Pasteur, Paris; CNR des virus des infections respiratoires, Institut Pasteur, Paris.

Martine Valette, Laboratoire de Virologie, CNR des virus des infections respiratoires, Institut des Agents Infectieux, Groupement Hospitalier Nord des HCL, Lyon.

EU-H study:

Odile Launay, Innovative clinical research network in vaccinology, I-REIVAC, CIC de Vaccinologie, Cochin-Pasteur, Paris

Marie Lachatre, I-REIVAC, CIC de Vaccinologie, Cochin-Pasteur, Paris

Nezha Lenzi, I-REIVAC

Anne-Sophie L'Honneur, Service de Virologie, Hôpital Cochin, Paris

Florence Galtier, I-REIVAC, Inserm CIC 1411 and Hôpital St Eloi, CHU de Montpellier

Olivier Gaget Olivier, Inserm CIC 1411 and Hôpital St Eloi, CHU de Montpellier

Vincent Foulongne, CHU de Montpellier

Corinne Merle, CHU de Montpellier

Philippe Vanhems, I-REIVAC, Paris, Hôpital Edouard Herriot, Lyon

Fabrice Lainé, I-REIVAC, Paris; CIC 1414, Hôpital Pontchaillou, Rennes

Gisèle Lagathu, laboratoire de virologie, CHU de Rennes

Fabrice Carrat, UPMC Univ Paris 06, IPLESP UMRS 1136, Public health department, Hôpital Saint-Antoine, Paris

EpiConcept

EU-PC and EU-H studies:

Marta Valenciano, Alain Moren, EpiConcept, Paris, France

EU-PC study:

Esther Kissling, EpiConcept, Paris, France

EU-H study:

Angela Rose, EpiConcept, Paris, France

European Centre for Disease Prevention and Control

Pasi Penttinen, ECDC, Stockholm, Sweden

Germany

EU-PC study:

Silke Buda, Department for Infectious Disease Epidemiology, Respiratory Infections Unit, Robert Koch Institute, Berlin

Ute Preuss, Department for Infectious Disease Epidemiology, Respiratory Infections Unit, Robert Koch Institute, Berlin

Kristin Tolksdorf, Department for Infectious Disease Epidemiology, Respiratory Infections Unit, Robert Koch Institute, Berlin

Barbara Biere, National Reference Center for Influenza, Robert Koch Institute, Berlin

Ralf Duerrwald, National Reference Center for Influenza, Robert Koch Institute, Berlin

Maria Smallfield, National Reference Center for Influenza, Robert Koch Institute, Berlin

Marianne Wedde, National Reference Center for Influenza, Robert Koch Institute, Berlin

Ireland

EU-PC study:

Lisa Domegan, HSE-Health Protection Surveillance Centre, Dublin; European Programme for Intervention Epidemiology Training (EPIET), European Centre for Disease Prevention and Control, (ECDC), Stockholm, Sweden

Meadhbh Hunt, HSE-Health Protection Surveillance Centre, Dublin

Joan O’Donnell, HSE-Health Protection Surveillance Centre, Dublin

Michael Joyce, Irish College of General Practitioners, Dublin

Olga Levis, Irish College of General Practitioners, Dublin

Claire Collins, Irish College of General Practitioners, Dublin

Linda Dunford, National Virus Reference Laboratory, University College Dublin

Joanne Moran, National Virus Reference Laboratory, University College Dublin

Grainne Tuite, National Virus Reference Laboratory, University College Dublin

Jeff Connell, National Virus Reference Laboratory, University College Dublin

Cillian de Gascun, National Virus Reference Laboratory, University College Dublin

Navarra

EU-H study:

Itziar Casado, Instituto de Salud Pública de Navarra, IdiSNA, CIBERESP, Pamplona

Leticia Fernandino, Instituto de Salud Pública de Navarra, IdiSNA, CIBERESP, Pamplona

Iván Martínez-Baz, Instituto de Salud Pública de Navarra, IdiSNA, CIBERESP, Pamplona

Regna Juanbeltz, Instituto de Salud Pública de Navarra, IdiSNA, CIBERESP, Pamplona

Carlos Gómez Ibañez, Instituto de Salud Pública de Navarra, IdiSNA, CIBERESP, Pamplona

Jesús Castilla, Instituto de Salud Pública de Navarra, IdiSNA, CIBERESP, Pamplona

Ana Navascués, Complejo Hospitalario de Navarra, IdiSNA, Pamplona

Aitziber Aguinaga, Complejo Hospitalario de Navarra, IdiSNA, Pamplona

Carmen Ezpeleta, Complejo Hospitalario de Navarra, IdiSNA, Pamplona

The Netherlands

EU-PC study:

Adam Meijer, National Institute for Public Health and the Environment (RIVM), Bilthoven

Wim van der Hoek, National Institute for Public Health and the Environment (RIVM), Bilthoven

Mariam Bagheri, National Institute for Public Health and the Environment (RIVM), Bilthoven

Sharon van den Brink, National Institute for Public Health and the Environment (RIVM), Bilthoven

Frederika Dijkstra, National Institute for Public Health and the Environment (RIVM), Bilthoven

Gabriel Goderski, National Institute for Public Health and the Environment (RIVM), Bilthoven

Marit de Lange, National Institute for Public Health and the Environment (RIVM), Bilthoven

Ton Marzec, National Institute for Public Health and the Environment (RIVM), Bilthoven

Pieter Overduin, National Institute for Public Health and the Environment (RIVM), Bilthoven

Elsa Poorter, National Institute for Public Health and the Environment (RIVM), Bilthoven

Daphne Reukers, National Institute for Public Health and the Environment (RIVM), Bilthoven

Anne Carola Teirlinck, National Institute for Public Health and the Environment (RIVM), Bilthoven

Lisa Wijsman, National Institute for Public Health and the Environment (RIVM), Bilthoven

Gé Donker, Nivel (the Netherlands Institute for Health Services Research), Utrecht

Mariette Hooiveld, Nivel (the Netherlands Institute for Health Services Research), Utrecht

Portugal

EU-PC study:

Verónica Gomez, Departamento de Epidemiologia, Instituto Nacional de Saúde Dr Ricardo Jorge, Lisbon

Irina Kislaya, Departamento de Epidemiologia, Instituto Nacional de Saúde Dr Ricardo Jorge, Lisbon

Ausenda Machado, Departamento de Epidemiologia, Instituto Nacional de Saúde Dr Ricardo Jorge, Lisbon

Baltazar Nunes, Departamento de Epidemiologia, Instituto Nacional de Saúde Dr Ricardo Jorge, Lisbon

Ana Paula Rodrigues, Departamento de Epidemiologia, Instituto Nacional de Saúde Dr Ricardo Jorge, Lisbon

Patrícia Conde, Departamento de Doenças Infeciosas, Instituto Nacional de Saúde Dr Ricardo Jorge, Lisbon

Inês Costa, Departamento de Doenças Infeciosas, Instituto Nacional de Saúde Dr Ricardo Jorge, Lisbon

Paula Cristóvão, Departamento de Doenças Infeciosas, Instituto Nacional de Saúde Dr Ricardo Jorge, Lisbon

Raquel Guiomar, Departamento de Doenças Infeciosas, Instituto Nacional de Saúde Dr Ricardo Jorge, Lisbon

Pedro Pechirra, Departamento de Doenças Infeciosas, Instituto Nacional de Saúde Dr Ricardo Jorge, Lisbon

Romania

EU-PC + EU-H:

Mihaela Lazar,”Cantacuzino” National Medico-Military Institute for Research and Development, Bucharest

Carmen Maria Cherciu,”Cantacuzino” National Medico-Military Institute for Research and Development, Bucharest

Maria Elena Mihai,”Cantacuzino” National Medico-Military Institute for Research and Development, Bucharest

Elena Stoian,”Cantacuzino” National Medico-Military Institute for Research and Development, Bucharest

Cerasella Dragomirescu,”Cantacuzino” National Medico-Military Institute for Research and Development, Bucharest

Mircea Ioan Popa,”Cantacuzino” National Medico-Military Institute for Research and Development, Bucharest

EU-H:

Gabriela Juganariu, Clinical Hospital of Infectious Diseases “Sf Parascheva”, Iasi

Maria Gradinaru, Clinical Hospital of Infectious Diseases “Sf Parascheva”, Iasi

Izabela Loghin, Clinical Hospital of Infectious Diseases “Sf Parascheva”, Iasi

Liliana Vlad, Clinical Hospital of Infectious Diseases “Sf Parascheva”, Iasi

Elena Duca, Clinical Hospital of Infectious Diseases “Sf Parascheva”, Iasi

Mihnea Hurmuzache, Clinical Hospital of Infectious Diseases “Sf Parascheva”, Iasi

Carmen Dorobăț, Clinical Hospital of Infectious Diseases “Sf Parascheva”, Iasi

Grațiela Tardei, Clinical Hospital of Infectious Diseases “Dr Victor Babes”, Bucharest

Violeta Melinte, Clinical Hospital of Infectious Diseases “Dr Victor Babes”, Bucharest

Mihaela Zaharia, Clinical Hospital of Infectious Diseases “Dr Victor Babes”, Bucharest

Maria Nica Clinical Hospital of Infectious Diseases “Dr Victor Babes”, Bucharest

Corneliu Petru Popescu, Clinical Hospital of Infectious Diseases “Dr Victor Babes”, Bucharest

Emanoil Ceausu, Clinical Hospital of Infectious Diseases “Dr Victor Babes”, Bucharest

Simin-Aysel Florescu, Clinical Hospital of Infectious Diseases “Dr Victor Babes”, Bucharest

Spain

The cycEVA work group:

Amparo Larrauri, National Centre of Epidemiology, Institute of Health Carlos III, CIBERESP

Alin Gherasim, National Centre of Epidemiology, Institute of Health Carlos III, CIBERESP

Clara Mazagatos, National Centre of Epidemiology, Institute of Health Carlos III, CIBERESP

Francisco Pozo, National Centre for Microbiology, National Influenza Reference Laboratory, WHO-National Influenza Centre, Institute of Health Carlos III

Inmaculada Casas, National Centre for Microbiology, National Influenza Reference Laboratory, WHO-National Influenza Centre, Institute of Health Carlos III

Luis García Comas, Dirección General de Salud Pública, Comunidad de Madrid

María Esther Insua Marisquerena, Dirección General de Salud Pública, Comunidad de Madrid

Juan Carlos Galán, Laboratorio Hospital Ramón y Cajal, CIBERESP

Mª Dolores Folgueira, Laboratorio Hospital Doce de Octubre

Jesús Castilla, Navarra Instituto de Salud Pública de Navarra - IdiSNA, Pamplona, CIBERESP

Manuel García Cenoz, Navarra Instituto de Salud Pública de Navarra - IdiSNA, Pamplona, CIBERESP

Ana Navascués, Complejo Hospitalario de Navarra

Carmen Quiñones Rubio, Dirección General de Salud Pública y Consumo de La Rioja

Eva Martinez Ochoa, Dirección General de Salud Pública y Consumo de La Rioja

Miriam Blasco, Laboratorio Hospital San Pedro de Logroño

Jaume Gimenez Duran, Servicio de Epidemiología, Dirección General de Salud Pública, Mallorca

Juana Maria Vanrell, Servicio de Epidemiología, Dirección General de Salud Pública, Mallorca

Jordi Reina, Laboratorio del Hospital Son Espases, Mallorca

Daniel Castrillejo, Servicio de Epidemiología. DGSC, Consejería de Bienestar Social y Sanidad, Ciudad Autónoma de Melilla

The Spanish Influenza Sentinel Surveillance System:

Amparo Larrauri, National Centre of Epidemiology, Institute of Health Carlos III, CIBERESP

Alin Manuel Gherasim, National Centre of Epidemiology, Institute of Health Carlos III, CIBERESP

Clara Mazagatos, National Centre of Epidemiology, Institute of Health Carlos III, CIBERESP

Concha Delgado, National Centre of Epidemiology, Institute of Health Carlos III, CIBERESP

Jesus Oliva, National Centre of Epidemiology, Institute of Health Carlos III, CIBERESP

Francisco Pozo, National Centre for Microbiology, National Influenza Reference Laboratory, WHO-National Influenza Centre, Institute of Health Carlos III

Inmaculada Casas, National Centre for Microbiology, National Influenza Reference Laboratory, WHO-National Influenza Centre, Institute of Health Carlos III

All the participants in the Spanish Influenza Surveillance System

The Spanish IMOVE hospital study:

Amparo Larrauri, National Centre of Epidemiology, Institute of Health Carlos III, CIBERESP

Alin Manuel Gherasim, National Centre of Epidemiology, Institute of Health Carlos III, CIBERESP

Clara Mazagatos, National Centre of Epidemiology, Institute of Health Carlos III, CIBERESP

Francisco Pozo, National Centre for Microbiology, National Influenza Reference Laboratory, WHO-National Influenza Centre, Institute of Health Carlos III

Inmaculada Casas, National Centre for Microbiology, National Influenza Reference Laboratory, WHO-National Influenza Centre, Institute of Health Carlos III

Miriam Latorre, Hospital Universitario Miguel Servet, Zaragoza

María Isabel Millán Lou, Hospital Universitario Miguel Servet, Zaragoza

Ana María Milagro, Hospital Universitario Miguel Servet, Zaragoza

Amaia Aizpurúa, Laboratorio Hospital Donostia, CIBERER

Gustavo Cilla, Laboratorio Hospital Donostia, CIBERER

Milagrosa Montes, Laboratorio Hospital Donostia, CIBERER

Sweden

EU-PC study:

Mia Brytting, The Public Health Agency of Sweden, Stockholm

Theresa Enkirch, The Public Health Agency of Sweden, Stockholm

Annasara Carnahan, The Public Health Agency of Sweden, Stockholm

Åsa Wiman, The Public Health Agency of Sweden, Stockholm

United Kingdom (UK)

UK study:

Richard Pebody, Public Health England, London

Heather Whitaker, Public Health England, London

Nick Andrews, Public Health England, London

Joanna Ellis, Public Health England, London

Diogo FP Marques, Health Protection Scotland, Glasgow

Simon Cottrell, Public Health Wales, Cardiff

Arlene Reynolds, Health Protection Scotland, Glasgow

Rory Gunson, West of Scotland Specialist Virology Centre, Glasgow

Samantha J Shepherd, West of Scotland Specialist Virology Centre, Glasgow

Catherine Thompson, Public Health England, London

Monica Galiano, Public Health England, London

Chris Robertson, University of Strathclyde, Glasgow

Mark O’Doherty, Public Health Agency Northern Ireland, Belfast

Katie Owens, Public Health England, London

Ivelina Yonova, University of Surrey, Guildford, Royal College of General Practitioners, London

Catherine Moore, Public Health Wales, Cardiff

Jillian Johnson, Public Health Agency Northern Ireland, Belfast

Simon de Lusignan, University of Surrey, Guildford, Royal College of General Practitioners, London

Jim McMenamin, Health Protection Scotland, Glasgow

Maria Zambon, Public Health England, London
